# Dental radiography and safety awareness: Insights from radiographers, dentists, and students in a cross-sectional study

**DOI:** 10.1371/journal.pone.0314884

**Published:** 2025-03-04

**Authors:** Moawia Gameraddin, Alaa Abdulkhalq Alessa, Hajar Sulaiman Aloufi, Shatha Dhaifallah Alzaidi, Awadia Gareeballah, Hassan Ibrahim Alsaedy, Kamal Alsultan, Mariam Khogaly Supair, Ahmed Ali Alharthi, Emadeldin Mohamed Mukhtar, Ali Abdelrazig, Magbool Alelyani

**Affiliations:** 1 Department of Diagnostic Radiology, College of Applied Medical Sciences, Taibah University, Al-Madinah Al-Munawwarah, Kingdom of Saudi Arabia; 2 Department of Diagnostic Radiology, Faculty of Radiology Science and Medical Imaging, Alzaiem Alazhari University, Khartoum, Sudan; 3 Medical Imaging Department, King Abdulaziz Medical City, Jeddah, Saudi Arabia; 4 Radiological Sciences Department, Al Ghad International Colleges for Applied Medical Sciences, Al-Madinah Al-Munawwarah, Saudi Arabia; 5 Al-Noor Specialist Hospital, Mecca, Kingdom of Saudi Arabia; 6 Department of Radiological Sciences, College of Applied Medical Sciences, King Khalid University, Abha, Saudi Arabia; 7 Diagnostic Radiography Technology (DRT) Department, Faculty of Nursing and Health Sciences, Jazan University, Jazan, Saudi Arabia; University of Puthisastra, CAMBODIA

## Abstract

**Background:**

Dental radiographs are crucial diagnostic tools for oral disorders, but their low-level radiation can pose health risks over time because of its cumulative effects.

**Objectives:**

This study aimed to assess awareness and attitudes regarding dental radiography and safety among dentists, radiographers, dentistry students, and radiography students in western Saudi Arabia.

**Methods:**

This cross-sectional survey study was conducted in the western region of Saudi Arabia. A total of 309 participants were included in the study. A questionnaire containing 13 multiple-choice questions was administered to dentists, radiographers, dentistry students, and radiography students. The Statistical Package for Social Sciences (SPSS 23) was used for data analysis.

**Results:**

The mean knowledge and attitude scores of the participants were 61.09% and 65.13%, respectively. The mean score knowledge of dentists was 6.8 out of 12, that of radiographers was 7.04 out of 12, that of radiography students was 6.42 out of 12, and that of dentistry students was 6.83 out of 12. The attitude score was 35.45 out of 55, for dentists; 36.21 out of 55, for radiographers; 36.54 out of 55, for radiography students; and 34.79 out of 55, for dentistry students. The knowledge score increased significantly in participants who had Ph.D. and M.Sc. degrees (p value =  0.008). There was no significant variation in knowledge regarding gender, age, area of employment, or experience (p value < .05). The participants exhibited a positive attitude toward dental radiographic examination and radiation safety, with no significant difference observed among the groups (p value > .05).

**Conclusion:**

This study indicates good awareness among dentists, radiographers, and students about dental radiography and safety, with higher education levels indicating greater awareness and a positive attitude. The safety and knowledge of dental radiography exposure vary among groups, highlighting the need for continuous education and standardized training to improve the understanding and usage of radiation hazards.

## Introduction

Dental radiography involves exposing teeth and structures to X-ray radiation to produce radiographic images [1,2]. These images are crucial for detecting lesions, diseases, and various conditions, as well as confirming suspected diseases, and localizing lesions. X-ray images provide critical information for dental professionals during procedures, evaluating growth and development, and documenting patient conditions. To reduce health risks, exposure should be kept as low as reasonably achievable (ALARA) [3–5].

According to previous reports, dentists, radiographers, dental students, and diagnostic radiology technology students need more knowledge and awareness about safety and protection from radiation effects and the practice of dental radiography [6]. Despite the critical role of dental radiography in diagnostic and therapeutic procedures, there is considerable variation in attitudes, knowledge, and safety practices among health care professionals and students involved in radiographic procedures. This inconsistency can lead to suboptimal patient care, increased radiation exposure risks, and a lack of standardized safety protocols.

In our region, more studies should be conducted on dental radiography assessment among health care professionals and students. Therefore, this study comprehensively examines and analyzes attitudes and knowledge about dental radiography among distinct cohorts, including radiographers, dentists, dentistry students, and diagnostic radiology technology students. The findings of this study will provide valuable insights into the current state of knowledge and attitudes toward dental radiography and safety. Additionally, this study will contribute to the development of more effective educational programs and policies to ensure the highest standards of radiographic safety in dental practice.

This study is vital for identifying critical areas where dental radiography knowledge and practice gaps exist. By understanding these gaps, educators and policymakers can develop more effective training programs and safety protocols, ultimately enhancing the quality of patient care and reducing radiation exposure risk. Therefore, this study aims to fill the knowledge gap and attitudes by conducting a thorough investigation to identify the prevalent attitudes and levels of awareness within these various groups.

## Materials and methods

This cross-sectional survey comprehensively examines and analyzes attitudes and knowledge about dental radiography and safety among distinct cohorts, including radiographers, dentists, dentistry students, and radiography students. The study was conducted from Apr 15 to May 2024. The population of the study included radiographers, dentists, dentistry students, and radiography students.

The inclusion criteria were dentists, radiographers, and graduate students in diagnostic radiology technology and dentistry in the western region of Saudi Arabia. The exclusion criteria were dentists, radiographers, and graduate students in diagnostic radiology technology and dentistry outside the western region of Saudi Arabia.

### Sampling and sample size

A total of 309 participants were selected to satisfy the sample size for this study. The sample size (n) was calculated via the sample size formula (n =  P (1-P) z2/d2). After considering a prevalence of 50% from previous studies, where d =  0.05 and z =  1.96, P is the population proportion, applying a confidence level of 95% and 80% power of the study.

### Data collection

The data were collected via a self-administered online survey distributed through social media and the radiology departments of hospitals in the western region of Saudi Arabia. The questionnaire’s content validity was approved by specialists with specialized knowledge in radiography and dentistry. The survey’s internal consistency reliability was assessed via Cronbach’s alpha, which yielded a score of 64.2%.

The survey contained two parts: 1) demographic details and 2) dependent variables, which included knowledge and attitudes data. There were 12 questions related to knowledge and 11 questions related to attitudes. The study variables included dependent variables such as participants’ attitudes and knowledge regarding dental radiologic examinations and safety. The independent variables were demographic characteristics such as age, sex (dichotomous variable), dentist, radiographer, dental student, radiography student, experience, area of work, and education level of the participants.

### Data analysis and interpretation

The data were analyzed via SPSS version 23, which employs suitable statistical tests to ascertain the significance and associations among the study variables. Qualitative data are presented as frequencies and percentages. The continuous variables (attitudes and knowledge scores) are expressed as the means ±  standard deviations (SDs). The chi-square test was applied for comparisons of qualitative data such as the responses of the participants to the provided questions. Owing to the nonnormal distribution of the knowledge and attitude scores, we employed nonparametric tests, specifically the Mann‒Whitney and Kruskal‒Wallis tests, to determine the relationships between the demographic variables and the average score levels. Each correct response was awarded 1 point, whereas incorrect responses received no points. The cumulative scores reflected the degree of knowledge for each respondent, with separate scores computed for each of the ten sections analyzed. Nominal knowledge levels were categorized as follows: good (greater than 50% correct answers), moderate (exactly 50% correct answers), and poor (less than 50% correct answers). For the second section on ‘attitude’, comprising 55 recorded responses, the median score was determined to be 36. Responses scoring above the median were categorized as ‘positive attitudes’, whereas those scoring at or below the median were categorized as ‘negative attitudes’. P values less than 0.05 were considered significant.

### Ethical considerations

The College of Applied Medical Sciences research ethics committee approved the study and provided the number 2024/184/306 DRD. The participants’ consent was collected before the start of the study, and the questionnaires were provided via Google® forms. The information submitted was kept strictly confidential for research reasons, and no participants’ identities were revealed.

## Results

### Demographic data

A total of 309 participants responded to the survey. The distributions of the gender and age groups of the participants and their demographic data are summarized in Table 1. The frequency in females was greater than that in males (173 vs. 136). A significant majority (85.4%) were between 21 and 30 years of age, indicating that the sample mainly included younger individuals. The largest group included those with a bachelor’s degree (48.5%), followed by undergraduate students (31.7%). Postgraduate and higher qualifications constituted a smaller percentage. The participants were evenly distributed among students in diagnostic radiology, dentistry, radiographers, and dentists, with each group comprising approximately one-third of the sample.

**Table 1:** Distribution of gender, age group, education level, designation, and experience of the participants.

Variables	Frequency	Percent
**Gender**		
Female	173	56.00
Male	136	44.00
**Age groups**		
21–30 years	264	85.40
31–40 years	45	14.60
**Education level**		
Undergraduate students	98	31.70
Interns	30	9.70
Postgraduate students	11	3.60
Bachelor (B.SC. or BDS)	150	48.50
Ph.D.	10	3.20
Fellowship	1	0.30
Master	9	2.90
**Designation**		
Radiography students	98	31.70
Dentistry students	57	18.40
Radiographers	56	18.10
Dentists	98	31.70
**Experience**		
None	143	46.30
1–5 year/s	136	44.00
6–9 years	21	6.80
Greater than 10 years	9	2.90

**Fig 1:**
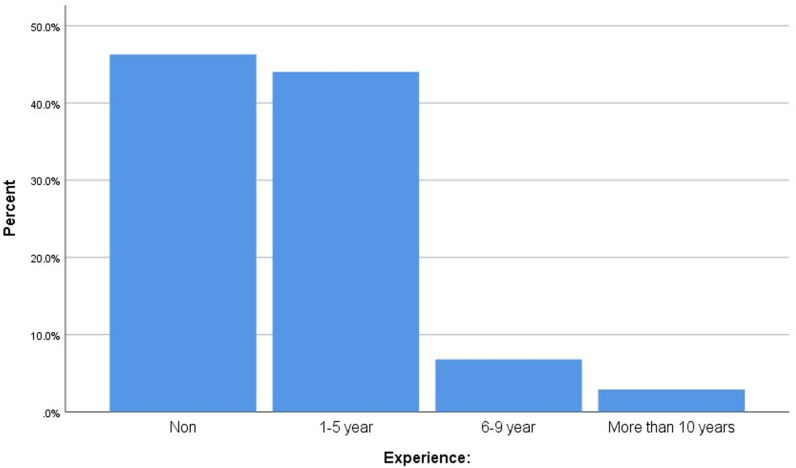
Distribution of experience among the study sample.

The distribution of participants experience is shown in Fig 1. Nearly half of the participants had no experience (46.3%), and a significant proportion (44%) had 1–5 years of experience. Only a small percentage had more than ten years of experience (2.9%).

**Fig 2:**
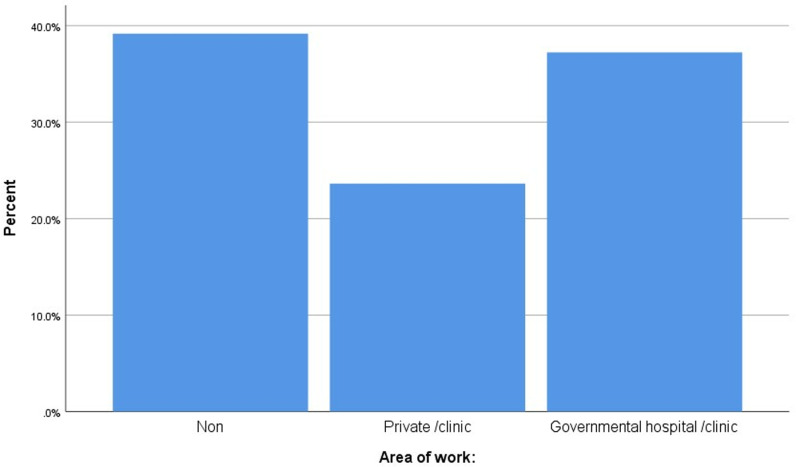
Distribution of the area of employment among the study sample.

The distribution of the employment region is shown in Fig 2. The majority (39.2%) had no work experience since they were students. There were more participants who worked in governmental hospitals or clinics than in private hospitals or clinics (37.2% vs. 23.6%).

### Knowledge and attitude scores

**Fig 3:**
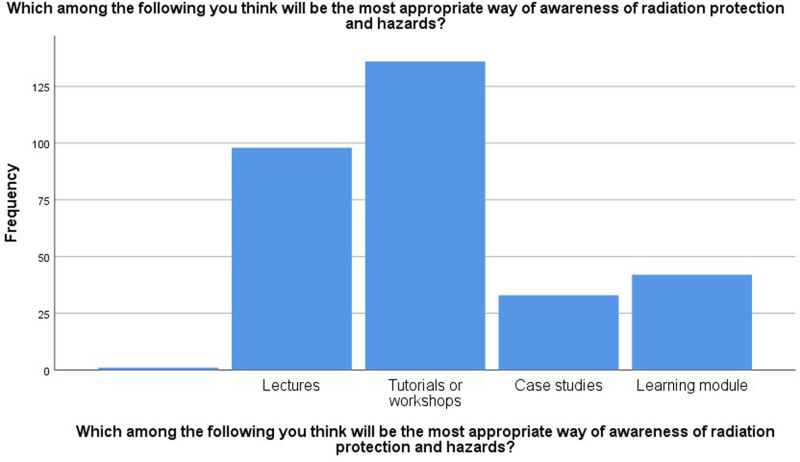
Responses of the participants to the most appropriate way of awareness for radiation protection and hazards.

The overall attitude and knowledge scores are shown in Table 2. The mean attitude score was 35.82, with a positive attitude (65.13%), while the knowledge score was 6.72 (61.01%), which was considered good. The participants’ responses to the most appropriate way to become aware of radiation protection and hazards are shown in Fig 3. Most responded that tutorials or workshops were the most appropriate tools for awareness of radiation hazards and protection (44%).

**Table 2:** The scores of knowledge and attitudes among the participants.

Domain	Minimum	Maximum	Mean	Median	SD	Evaluation
Attitude	24.00	50.00	35.82	36.00	4.48	65.13%
Knowledge	4.00	11.00	6.73	7.00	1.66	61.09%

The knowledge domain for every question is presented in Table 3. The responses were approximately split evenly, with 49.2% correctly acknowledging the danger, 58.1% correctly indicating that the room’s walls could reflect X-rays, and 41.6% of the participants responded incorrectly. A total of 52.9% of the participants understood the ALARA (as low as reasonably achievable) principle, and the rest did not. Notably, 38.5% of the participants reported that the recommended standing distance for an operator during dental radiography exposure was 6 feet and 90–135 degrees. With respect to awareness of dental radiography, 80.9% of the participants were aware of cone beam CT (CBCT), intraoral exams (77.5%), orthopantomography (OPG) (68.5%), and dosimetry (84.1%).

**Table 3:** Participants’ knowledge of dental radiography and safety.

Questions	Responses	Frequency	Percent	Mean	Median	SD
Do you believe that dental X-rays are dangerous?	No	157	50.80	0.49	0.00	0.50
	Yes (correct)	152	49.20			
It is possible for the room’s walls to reflect X-rays?	Yes	129	41.70	0.58	1.00	0.49
	No (correct)	180	58.30			
Does 20 mSv is the annual radiation dose limit for a dentist?	Yes	120	38.80	0.61	1.00	0.49
	No (correct)	189	61.20			
Do you know cone beam CT (CBCT)?	Yes (correct)	250	80.90	0.81	1.00	0.39
	No	59	19.10			
Is it permissible for individuals to enter the room while an exposure is in progress?	Yes	243	78.60	0.21	0.00	0.41
	No (correct)	66	21.40			
Dosimeter is used to measure the radiation dose.	Yes (correct)	260	84.10	0.84	1.00	0.37
	No	49	15.90			
What is the radiation exposure ALARA principle?	As low as radiation as possible	85	27.40	0.53	1.00	0.50
	As low rates as possible	10	3.30			
	As low as reasonably achievable (correct)	164	52.90			
	I don’t know	50	16.10			
What is the recommended standing distance for an operator during dental radiography exposure?	4 feet and 90–135 degrees	76	24.50	0.39	0.00	0.49
	4 feet and 60–90 degrees	66	21.30			
	6 feet and 90–135 degrees (correct)	119	38.50			
	6 feet and 60–90 degrees	48	15.50			
In dental radiography, which organ holds the highest priority for protection?	Gonads	25	8.10	0.83	1.00	0.38
	Thyroid	257	830			
	Skin	17	5.50			
	Bone marrow	10	3.20			
Are you aware of any advancements in dental radiography techniques that aim to reduce radiation exposure for patients?	Yes, I’m aware of specific advancements (correct)	104	33.50	0.34	0.00	0.47
	I know there have been advancements, but I’m not sure of the details	136	44.00			
	No, I’m not aware of any advancements	69	22.30			
What is occlusal dental radiography?	Intraoral dental exam (correct)	240	77.50	0.78	1.00	0.42
	Extraoral dental exam	69	22.30			
What is Orthopantomography (OPG)?	Intraoral dental exam	97	31.30	0.31	0.00	0.47
	Extraoral dental exam (correct)	212	68.50			

Notably, 78.6% responded that it is permissible for individuals to enter the room during exposure, whereas only 21.4% responded that it is not permissible. Eighty-three percent knew that the thyroid was a sensitive organ needing protection during dental exams.

Table 4 presents the results of an assessment of dental radiography and safety awareness among four study groups: radiography students, dentistry students, radiographers, and dentists. The responses to several questions highlight varying knowledge and misconceptions across these groups. Dentists were more aware than radiographers regarding the hazard of X-ray radiation (54.1% vs. 48.2%). In comparison, dentistry students were more aware than radiography students were (61.4% vs. 37.8%), with significant differences among the groups (P value =  0.023). This finding underscores the urgent need for improved education on radiation safety among radiography students and radiographers and the importance of standardized training in dental radiography.

**Table 4:** Assessment of awareness of dental radiography and safety among the study groups.

Questions	Responses	Radiography students N (%)	Dentistry students N (%)	Radiographers N (%)	Dentists N (%)	P value
Do you believe that dental X-rays are dangerous?	No	61 (62.2%)	22 (38.6%)	29 (51.8%)	45 (45.9%)	0.023
	Yes (correct)	37 (37.8%)	35 (61.4%)	27 (48.2%)	53 (54.1%)	
The room’s walls can reflect X-rays.	Yes	46 (46.9%)	18 (31.6%)	23 (41.1%)	42 (42.9%)	0.312
	No (correct)	52 (53.1%)	39 (68.4%)	33 (58.9%)	56 57.1%	
Does 20 mSv in the annual radiation dose limit a dentist?	Yes	39 (39.8%)	23 (40.4%)	21 (37.5%)	37 (37.8%)	0.980
	No (correct)	59 (60.2%)	34 (59.6%)	35 (62.5%)	61 (62.2%)	
Do you know cone beam CT (CBCT)?	Yes (correct)	59 (60.2%)	53 (93.0%)	46 (82.1%)	92 (93.9%)	0.39
	No	39 (39.8%)	4 (7.0%)	10 (17.9%)	6 (6.1%)	
Is it permissible for individuals to enter the room while an exposure is in progress?	Yes	83 (84.7%)	45 (78.9%)	42 (75.0%)	73 (74.5%)	0.310
	No (correct)	15 (15.3%)	12 (21.1%)	14 (25.0%)	25 (25.5%)	
A dosimeter is used to measure the radiation dose.	Yes (correct)	89 (90.8%)	46 (80.7%)	50 (89.3%)	75 (76.5%)	0.027
	No	9 (9.2%)	11 (19.3%)	6 (10.7%)	23 (23.5%)	
What is the radiation exposure ALARA principle?	Correct	68 (69.4%)	25 (43.9%)	36 (64.3%)	35 35.7%	< 0.001
	Incorrect	30 (30.6%)	32 (56.1%)	20 (35.7%)	63 (64.3%)	
What is the recommended standing distance for an operator during dental radiography exposure?	Correct	30 (30.6%)	31 (54.4%)	17 (30.4%)	41 (41.8%)	0.014
	Incorrect	68 (69.4%)	26 (45.6%)	39 (69.6%)	57 (58.2%)	
In dental radiography, which organ holds the highest priority for protection?	Correct	85 (86.7%)	41 (71.9%)	45 (80.4%)	86 (87.8%)	0.05
	Incorrect	13 (13.3%)	16 (28.1%)	11 (19.6%)	12 (12.2%)	
Are you aware of any advancements in dental radiography techniques that aim to reduce patient radiation exposure?	Aware	27 (27.6%)	18 (31.6%)	26 (46.4%)	33 (33.7%)	0.120
	Not aware	71 (72.4%)	39 (68.4%)	30 (53.6%)	65 (66.3%)	
What is occlusal dental radiography?	Intraoral dental exam (correct)	72 (73.5%)	44 (77.2%)	42 (75.0%)	82 (83.7%)	0.352
	Extraoral dental exam	26 (26.5%)	13 (22.8%)	14 (25.0%)	16 (16.3%)	
What is Orthopantomography (OPG)?	Intraoral dental exam	36 (36.7%)	11 (19.3%)	23 (41.1%)	27 (27.6%)	0.041
	Extraoral dental exam (correct)	62 (63.3%)	46 (80.7%)	33 (58.9%)	71 (72.4%)	

Another notable result is understanding the radiation exposure ALARA principle. Radiography students (69.4%) had the highest level of awareness, whereas dentists (35.7%) had the lowest correct response rate, with a significant difference among the groups (P value <  0.001). This finding indicates a critical gap in ongoing professional education for practicing dentists. With respect to the use of dosimeters, radiography students (90.8%) and radiographers (89.3%) reported significantly greater awareness (P value =  0.027) than did dentists (76.5%). This highlights the potential for enhancing radiation monitoring practices within dental practices, emphasizing the need for improvement in this area.

The study also revealed that the understanding of certain imaging modalities, such as CBCT and OPG, varies among groups. Dentistry students were highly aware (93.0% and 80.7%, respectively), whereas radiography students and radiographers were less aware. This disparity underscores the necessity for targeted educational interventions to bridge knowledge gaps and ensure consistent safety practices across all dental professionals.

Overall, there was significant variation among the groups regarding the understanding of radiation hazards, usage of dosimetry, the ALARA principle, and the recommended standing distance during dental radiography exposure. The findings underscore the importance of continuous education and standardized training in dental radiography to enhance safety and knowledge across various professional groups within dentistry.

The mean knowledge score was significantly greater in males than in females (mean ranks 155.03 vs. 154.96, p value =  0.994), as depicted in Table 5. The education level significantly impacts knowledge (P value =  0.019). The level of knowledge increased significantly among the qualified participants (P value <  0.05). The undergraduate students had more knowledge than the interns did, postgraduates, and those with bachelor’s degrees. There were no significant differences among the groups regarding age, designation, experience, or area of work on knowledge of dental exams and safety (P value >  0.05), as shown in Table 5.

**Table 5:** Comparisons of knowledge among the participants.

Variables	Median	Frequency	Mean rank	P values
**Gender**				
Female	7.00	173	155.03	0.994
Male	7.00	136	154.96	
**Age**				
21–30 years	7.00	264	153.98	0.620
31–40 years	7.00	45	160.99	
**Education level**				
Undergraduate students	7.00	98	162.23	0.019
Interns	7.00	30	144.50	
Postgraduate students	6.00	11	138.82	
Bachelor (B.SC. or BDS)	7.00	150	145.29	
Ph.D.	7.00	10	191.80	
Fellowship	--	1	282.00	
Master	8.00	9	237.78	
**Designation**				
Diagnostic radiology students	6.00	98	139.38	0.175
Dentistry students	7.00	57	164.09	
Radiographers	7.00	56	167.34	
Dentists	7.00	98	158.29	
**Experience**				
None	7.00	143	152.39	0.192
1–5 year/s	7.00	136	151.56	
6–9 years	7.00	21	170.90	
Greater than 10 years	7.78	9	211.39	
**Area of work**				
None	7.00	121	150.83	0.799
Private/clinic	7.00	73	157.55	
Governmental hospital/clinic	7.00	115	157.77	

Fig 3 depicts the frequency of various educational methods used in the study, highlighting a significant preference for tutorials or workshops, which were mostly utilized by the participants (125 of 309). Lectures were also featured prominently, with a frequency slightly above 100. In contrast, case studies and learning modules were employed less frequently, with case studies and learning modules being used by approximately 30 participants. This distribution suggests a greater reliance on interactive and hands-on learning experiences, such as tutorials or workshops and lectures, over more independent or applied learning methods such as case studies and learning modules.

### Attitude assessment

The participants’ attitudes regarding dental radiography and safety are shown in Table 6. The attitude toward radiation protection and hazards was 74.4%, which was considered a positive attitude. In terms of whether X-rays are contraindicated for pregnant women, 50.5% of the participants agreed, which was considered a negative attitude. A total of 68.3% of the participants agreed that digital radiography requires less exposure than conventional radiography does, and the rest of the participants were neutral or disagreed. Notably, 29.1% of the participants agreed that CBCT has a lower dose than the panoramic machine does, and 53.4% of the participants disagreed. A total of 56% of the participants agreed that they obtained several radiographs to confirm a diagnosis, while 26.2% were neutral and 17.5% disagreed.

**Table 6:** Comparisons of attitude scores among the participants.

Variables	Median	Frequency	Mean rank	P values
**Gender**				
Female	36	173	154.59	0.928
Male	36	136	155.52	
**Age**				
21–30 years	36	264	155.31	0.883
31–40 years	36	45	153.19	
**Education level**				
Undergraduate students	36.00	98	159.84	0.953
Interns	35.50	30	156.08	
Postgraduate students	35.00	11	143.45	
Bachelor (B.SC. or BDS)	35.00	150	150.70	
Ph.D.	36.5	10	174.75	
Fellowship	--	1	201.00	
Master	36.00	9	157.50	
**Designation**				
Diagnostic radiology students	37	98	169.77	0.133
Dentistry students	35	57	135.85	
Radiographers	36.00	56	156.52	
Dentists	36	98	150.50	
**Experience**				
None	35.00	143	158.37	0.321
1–5 year/s	35.50	136	148.70	
6–9 years	37.00	21	175.62	
Greater than 10 years	36.00	9	148.50	
**Area of work**				
None	36.00	121	161.37	0.527
Private/clinic	35.00	73	146.71	
Governmental hospital/clinic	35.00	115	153.56	

The attitudes regarding the performance of dental exams by radiographers were positive, with 28.2% strongly agreeing and 37.2% agreeing, for a total of 65.4%. On the other hand, attitudes regarding the performance of dental exams by dentists were negative, with 16.8% strongly agreeing and 27.8% agreeing, for a total of 44.6%. The attitude regarding the hazard of radiation dose was positive, and the usage of lead aprons was negative.

### Comparisons of attitude scores among the participants

The study highlights varying knowledge and attitudes toward dental radiography and safety among different groups. The comparisons of attitudes among the participants are shown in Table 6. There were no significant differences in attitude scores between genders or age groups (P value >  0.05). Moreover, education level did not significantly affect the attitude scores. Slight differences were noted in the attitude scores among the different designations, with students in diagnostic radiology having slightly higher scores. Experience and area of work did not significantly impact attitude scores.

Males had greater attitudes than females did regarding dental radiography exams and radiation safety, but the difference was not significant (mean rank: 154.59 vs. 155.52; P value =  0.928). Interns had greater positive attitudes than undergraduates and postgraduates did, but the difference was not significant (P value <  0.05). The participants with Ph.D.s (mean rank =  191.8) and master’s degrees (mean rank =  237.78) had significantly greater mean score attitudes than did the other participants (P value =  0.019). Years of experience and area of work had no significant effect on attitudes (p values <  0.05). The study highlights varying knowledge and attitudes toward dental radiography and safety among different groups.

## Discussion

Radiological examinations are essential to clinical dental treatment, providing significant advantages to professionals and patients. Their duties include diagnosis, treatment planning, treatment guidance, prognosis prediction, and outcome monitoring. Dentists and radiographers should be knowledgeable about radiation to protect themselves and their patients. The current study investigated the knowledge and attitudes regarding dental examination and radiation safety among radiographers, dentists, radiography students, and dentistry students.

The demographic characteristics of the study participants reveal a predominantly young sample, with 85.4% of participants between 21 and 30 years old, which aligns with previous studies focusing on students and early-career professionals in health care settings. The gender distribution in our study (56% female and 44% male) reflects the increasing participation of females in health-related professions, a trend observed globally in similar research contexts. Comparatively, studies by Abuelhia et al. and Basheer et al. reported similar gender distributions in their surveys of health care professionals, highlighting the trend toward gender parity in this field [7,8].

The current study revealed that the average knowledge score of dental radiographic exams and radiation safety among the participants was 61.01%. The study revealed that the mean score of knowledge of radiographers was greater than that of dentists and dentistry and radiography students. In contrast, Furmaniak et al. reported that dentists’ mean score knowledge was greater than that of radiographers [9]. Another study reported that the mean score knowledge among Egyptian dentists regarding radiation safety was 56.9, which is lower than our finding. Our results revealed that awareness among dentistry students was greater than that among radiography students. Furmaniak et al. reported that radiography students’ knowledge scores were higher than those of dentistry students [9]; our results contradict this finding. There was no significant difference among the four groups. On the other hand, the study revealed that males had greater knowledge than females do regarding dental radiographic findings and radiation safety, although without significant variation.

A total of 49.2% of the participants reported that X-rays were hazardous, which needs to be improved. In contrast, Almohaimede et al. reported an 80.1% level of knowledge. They reported that 56% of the participants were aware of the harmful effects of radiation exposure [10]. A study performed by Shujalpurwala reported that knowledge and awareness regarding radiation hazards and protection among dentists were fair and adequate. Awareness of radiation hazards is essential for protecting patients and staff [11]. ALARA is the underlying principle that underpins radiation protection and improves the knowledge of radiation protection. The present study revealed that 52.9% of the participants were aware of the ALARA principle. A study revealed a percentage of 68.6%, which is higher than our finding [11]. Elmurabit et al. assessed knowledge and practices among Moroccan dentistry specialists in a cross-sectional study and reported that awareness of ALARA among participants was 41.6% [12]. Radiography students were more knowledgeable than dentistry students were, and radiographers were more aware than dentists were, without significant differences among the groups. Our findings showed that participants’ awareness of radiation dangers and protection could be improved. Awareness of the ALARA principle is a unique consideration for medical exposure to radiation.

With respect to the annual radiation dose limit for dentists, 38.8% were aware of this point, which is considered poor knowledge. In contrast, a previous study reported that the level of knowledge was 87.5%, which is higher than our findings. The mean knowledge score of the dentistry students in our study was 6.82 out of 12 (56.8%), which was higher than that of the dentistry students (53.3%). A previous study revealed that the knowledge dentistry students had regarding radiation protection and practice could have improved [13,14].

With respect to organ protection, knowledge regarding the protection of the thyroid gland was excellent. A previous study consistently reported that awareness of protecting the thyroid gland was high among dentists [12,15]. Recently, the ADA recommended that thyroid collars be no longer recommended for any dental imaging modality [16].

With respect to knowledge of dental radiographic examinations, the study revealed that the participants were highly aware of occlusal radiography (78%). Dentists were more knowledgeable than radiographers and dentistry and radiography students. A previous study revealed that dentists were moderately aware of occlusal radiography [17]. These findings indicate that dentists might have received the necessary education on these investigations.

The knowledge of the OPG among the participants improved. The knowledge of dentists was greater than that of radiographers but less than that of radiography students. A study by Ardakani and Sarayesh consistently reported that the level of knowledge regarding OPGs was good [17]. On the other hand, the awareness of CBCT was excellent; dentists were more aware of CBCT than radiographers were. Dentists are highly aware of CBCT, indicating an interest in this technique [17]. Radiography students were more aware of CBCT than dentistry students were. A previous study revealed that only 63.3% of dental students had heard of CBCT [18]. Therefore, efforts should be made to improve students’ awareness of CBCT, and the dentistry and radiography curriculums in schools should allocate more time to this new technology.

The participants’ attitudes toward the dental examination and radiation safety were good and were considered positive. Similarly, Mahasneh et al. reported that the average attitude toward quality assurance in dental radiography was good, without statistically significant variations across the study groups [19]. The attitudes of interns were found to be greater than those of undergraduate and postgraduate students. This finding is consistent with that of Mukta et al., who reported that attitudes were greater among interns than among medical students [20]. The attitudes of those with master’s degrees and Ph.D. degrees were greater than those with lower degrees.

Radiography students had greater attitudes than dentistry students, dentists, and radiographers did, but these differences were not significant among the groups. The attitude of dentists in this study was 35.46 out of 55 (64.5%), which is approximately similar to that reported by Yurt et al., who reported a mean attitude score of 8.3 ±  2.1 out of 27.97% dentists [15]. There were no significant variations in the attitude scores regarding sex, age, years of experience, or region of employment.

This study has certain limitations because the number of respondents could not be controlled and was not equally distributed. The heterogeneous presentation of the sample is a great challenge since it creates unequal numbers of participants among the groups. Furthermore, the number of responses varied by specialty and employment industry and was not evenly distributed.

## Conclusion

The participants demonstrated good knowledge and positive attitudes toward dental radiographic examinations and radiation safety. Dentistry and radiography students were more knowledgeable, with no significant differences. The safety and knowledge of dental radiography exposure vary among groups, highlighting the need for continuous education and standardized training to improve the understanding and usage of radiation hazards, focusing on the ALARA principle and suggesting that future research explore targeted interventions.

## Supporting information

S1 File Dental safet study. (XLSX)
