# Correction: Dental radiography and safety awareness: Insights from radiographers, dentists, and students in a cross-sectional study

**DOI:** 10.1371/journal.pone.0329955

**Published:** 2025-08-06

**Authors:** Moawia Gameraddin, Alaa Abdulkhalq Alessa, Hajar Sulaiman Aloufi, Shatha Dhaifallah Alzaidi, Awadia Gareeballah, Hassan Ibrahim Alsaedi, Kamal Alsultan, Mariam Khogaly Supair, Ahmed Ali Alharthi, Emadeldin Mohamed Mukhtar, Ali Abdelrazig, Magbool Alelyani

The sixth author’s name is spelled incorrectly. The correct name is: Hassan Ibrahim Alsaedi. The correct citation is: Gameraddin M, Alessa AA, Aloufi HS, Alzaidi SD, Gareeballah A, Alsaedi HI, et al. (2025) Dental radiography and safety awareness: Insights from radiographers, dentists, and students in a cross-sectional study. PLoS ONE 20(3): e0314884. https://doi.org/10.1371/journal.pone.0314884
